# Impact of Laparoscopic Sleeve Gastrectomy on Gastrointestinal Motility

**DOI:** 10.1155/2018/4135813

**Published:** 2018-04-05

**Authors:** Eleni Sioka, George Tzovaras, Konstantinos Perivoliotis, Vissarion Bakalis, Eleni Zachari, Dimitrios Magouliotis, Vassiliki Tassiopoulou, Spyridon Potamianos, Andreas Kapsoritakis, Antigoni Poultsidi, Konstantinos Tepetes, Constantine Chatzitheofilou, Dimitris Zacharoulis

**Affiliations:** Department of Surgery, University Hospital of Larissa, Larissa, Greece

## Abstract

**Objective:**

Laparoscopic sleeve gastrectomy (LSG) was considered mainly as a restrictive procedure due to anatomic alterations in the upper gastrointestinal tract. Additionally, due to neurohormonal alterations, LSG modifies the gastrointestinal motility, which controls appetite and feeling of satiety.

**Aim:**

The aim of the study was to review the impact of laparoscopic sleeve gastrectomy on gastrointestinal motility.

**Material and Methods:**

A search of the medical literature was undertaken in Pubmed, Web of Science, and Cochrane library. Esophageal, gastric, bowel motility were assessed separately.

**Results:**

Nine studies assessed esophageal motility. The data remain debatable attributing to the heterogeneity of follow-up timing, surgical technique, bougie size, and distance from pylorus. The stomach motility was assessed in eighteen studies. Functionally, the sleeve was divided into a passive sleeve and an accelerated antrum. All scintigraphic studies revealed accelerated gastric emptying after LSG except of one. Patients demonstrated a rapid gastroduodenal transit time. The resection of the gastric pacemaker had as a consequence aberrant distal ectopic pacemaking or bioelectrical quiescence after LSG. The bowel motility was the least studied. Small bowel transit time was reduced; opposite to that the initiation of cecal filling and the ileocecal valve transit was delayed.

**Conclusion:**

Laparoscopic sleeve gastrectomy has impacts on gastrointestinal motility. The data remain debatable for esophageal motility. Stomach and small bowel motility were accelerated, while the initiation of cecal filling and the ileocecal valve transit was delayed. Further pathophysiological studies are needed to evaluate the correlation of motility data with clinical symptoms.

## 1. Introduction

Laparoscopic sleeve gastrectomy (LSG) is the most frequently performed procedure in the world and has overtaken the “gold standard” Roux-en-Y gastric bypass (RYGB), which remains the most performed bariatric/metabolic procedure only in Latin/South America. The trend analysis demonstrated that LSG had the largest average annual percentage increase of approximately 9% from 2013 of the worldwide bariatric/metabolic surgical procedures [1]. Laparoscopic sleeve gastrectomy is positioned between the adjustable gastric banding and RYGB in terms of morbidity with effectiveness comparable to RYGB even at five years [2, 3].

Laparoscopic sleeve gastrectomy involves the removal of the gastric fundus and greater curvature portion of the stomach, leaving only a lesser curvature tube. Yehoshua et al. showed that after the sleeve, the fundus, which is the most distensible part of the stomach, is removed, thus leaving a sleeve which is characterized by markedly lesser distensibility and high intraluminal pressure, leading to the feeling of early satiety [4]. The abstraction of the fundus is related to physiological alterations in the upper gastrointestinal tract, since the angle of His and the receptive relaxation mechanism is abolished, and the gastric pacemaker is abandoned. Furthermore, the antrum is preserved in some cases depending on the distance of the resection, and the pylorus remains intact. These physiological changes are expected to provoke motility alterations at the gastrointestinal tract.

The aim of the study was to review the impact of laparoscopic sleeve gastrectomy on gastrointestinal motility.

## 2. Material and Methods

### 2.1. Search Strategy

A search of the medical literature was undertaken in Pubmed, Web of Science, and Cochrane library until August 2017. The keywords used were “sleeve gastrectomy” AND (motility OR gastrointestinal motility OR esophageal motility OR gastric motility OR bowel motility OR emptying OR manometry).

### 2.2. Eligibility Criteria

#### 2.2.1. Types of Studies

All studies assessing the impact of LSG on gastrointestinal motility were included. Esophageal, stomach, and bowel motilities were assessed separately.

#### 2.2.2. Types of Participants

The types of participants were obese patients (BMI > 30) undergoing laparoscopic sleeve gastrectomy.

#### 2.2.3. Types of Outcome Measures

The outcomes measured were esophageal, gastric, and bowel motility.

### 2.3. Inclusion and Exclusion Criteria

Two independent reviewers (Eleni Sioka and Konstantinos Perivoliotis) screened abstracts, reviewed full text versions of all studies classified, and extracted data. Any trial considered relevant was retrieved for further review. The full texts were independently assessed by two reviewers. Disagreements were resolved with a third reviewer. Only published articles in the English language were included. Meta-analysis, systematic reviews, letters to the editor, case studies, non-English language publications, duplicate studies, experimental studies, and conference papers were excluded.

### 2.4. Data Extraction and Quality Assessment

One reviewer (Konstantinos Perivoliotis) extracted data from selected trials and a second reviewer (Eleni Sioka) checked for accuracy. The data were standardized and extracted from each study, and data were recorded into a database. The variables collected were first author, year of publication, country, type of study, number of participants, method of assessment, timing points, and main findings.

## 3. Results

### 3.1. Study Selection

**Figure 1:**
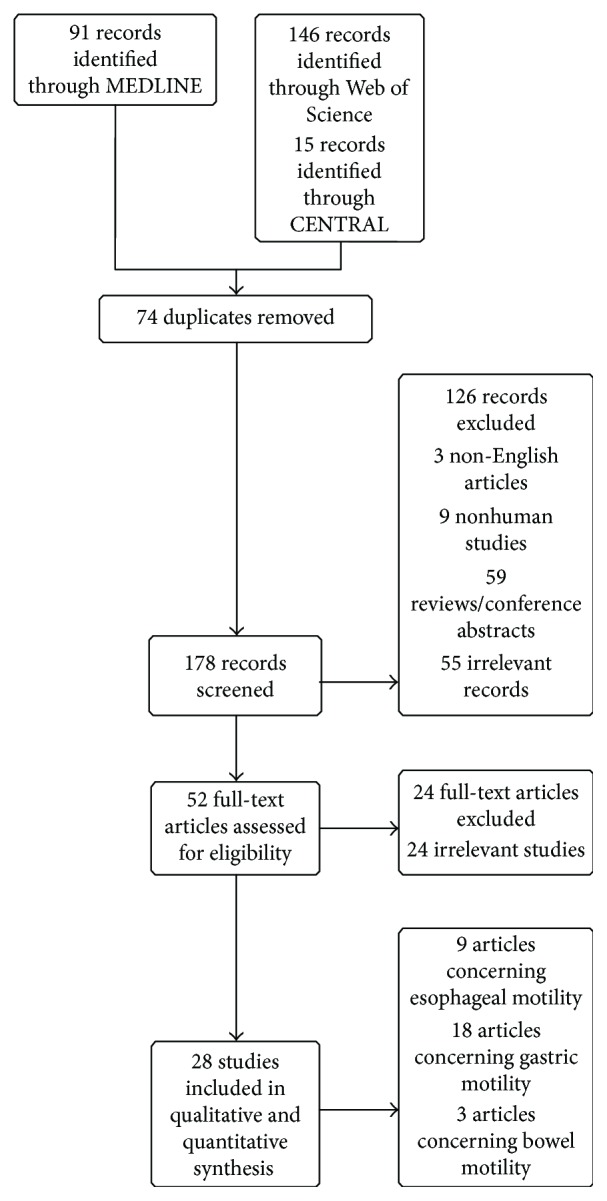
Flow diagram.

One hundred seventy eight studies were screened for eligibility and fifty two studies assessed in full text. Twenty four studies were excluded due to various reasons. Finally, 28 studies were eligible to be included in the review (Figure 1). There was complete agreement among the authors as to the inclusion of these studies.

### 3.2. Esophageal Motility

Nine studies assessed esophageal motility. Two studies were conducted in Greece [5, 6], one in France [7], two in Italy [8, 9], one in Argentina [10], one in Netherlands [11], one in Germany [12], aand one in Chile [13]. All except of one [7] were prospective studies.

The postoperative follow-up ranged from 6 days to 50 months. The bougie size ranged from 32 to 40F. The distance from the pylorus ranged from 2 to 6 cm. The lower esophageal sphincter (LES) length was reduced in three studies [5, 9, 13] increased in two studies [6, 10], and unchanged in one study [8]. The LES resting pressure decreased in four studies [5, 10, 11, 13], increased in two studies [6, 12], and remained unchanged in two studies [8, 9]. The amplitude pressure was reduced in two studies [5, 11], increased in one study [10], and unchanged in one study [8]. Amplitude pressure decreased in distal esophagus and increased in the other parts in one study [6]. Ineffective motility was reported in four studies. The percentage was increased from 10% to 45% postoperatively in one study [9], while it was the same preoperatively (7%) in another study [10], was increased at 5% (1/20 patients) in one study [11], and was reported at 37.7% (20/53 patients) in another study [7]. The percentage of normal peristaltic contractions was not affected in one study [11], while it increased in another study [5]. The summary of studies is reported in Table 1.

### 3.3. Stomach Motility

Eighteen studies assessed stomach motility. Eleven studies were conducted in Europe (Spain [*n* = 2] [14, 15], Italy [*n* = 3] [16–18], Greece [*n* = 3] [19–21], Czech Republic [*n* = 1] [22], Germany [*n* = 1] [23], and the Netherlands [*n* = 1] [24]), whereas one study was performed in the USA [25], two studies in India [26, 27], one study in New Zealand [28], one in Egypt [29], one in Chile [30], and one in Israel [31]. Twelve studies were prospective studies, four studies were prospective randomized controlled studies [14, 17, 22, 27], and two were retrospective studies [18, 25]. The method of gastric emptying assessment was gastric scintigraphic studies in fourteen studies and magnetic resonance imaging (MRI) in one study [23], while one study assessed the gastric slow-wave pacemaking using laparoscopic high-resolution (HR) electrical mapping [28], and two studies assessed the gastroduodenal transit time by radiological upper gastrointestinal series [18, 25].

The postoperative follow-up ranged from 3 to 24 months. The bougie size ranged from 32 to 48F. The distance from the pylorus ranged from 2 to 8 cm. All studies showed accelerated gastric emptying after LSG except for one [31]. Functionally, the sleeve was divided into a passive sleeve and an accelerated antrum. The patients demonstrated a rapid gastroduodenal transit time. The resection of the gastric pacemaker had as a consequence aberrant distal ectopic pacemaking or bioelectrical quiescence after LSG. No correlation was observed between gastric emptying and postprandial symptoms in one study. No correlation was found between gastroduodenal transit time and weight loss in one study [25], while in another study, patients showing a rapid gastroduodenal transit had better weight loss than patients presenting with a slow voiding rate [18]. The summary of the studies are presented in Table 2.

### 3.4. Bowel Motility

Three studies assessed the bowel motility. Two were prospective studies and one was prospective randomized study. One study was conducted in Japan, one in Greece, and one in India [21, 27, 32]. Trung et al. evaluated the intestinal motility using cine MRI preoperatively and 3 months postoperatively and found reduced intestinal transit time leading to accelerated intestinal motility [32]. Melissas et al. evaluated prospectively 21 patients preoperatively with a g-camera and 4 months postoperatively. Apart from the acceleration of small bowel transit time, the authors showed that initiation of cecal filling and the ileocecal valve transit was delayed [21]. Similarly, Shah et al. showed decreased small bowel transit time after LSG [27] (Table 3).

## 4. Discussion

The aim of this study was to review the impact of laparoscopic sleeve gastrectomy on gastrointestinal motility. The gastrointestinal motility research might be essential to recognize the behavior of the created sleeve, to explain possible clinical symptoms-implications, and to elucidate possible mechanisms of actions of this procedure. According to the Second International Consensus Summit for Sleeve Gastrectomy, the panel of experts voted the following as mechanisms of action: restriction: 79%, gastric emptying: 0%, hormonal: 16%, malabsorption: 0%, and other: 3% [33]. Since 2007, LSG was characterized as more than a restrictive procedure [19]. Additional supportive evidence came from studies assessing hormonal alterations after LSG. Concerning hormonal changes, it seems that patients experienced a pronounced and long-lasting decrease of circulating ghrelin and increased postprandial release of the CCK, GLP-1, and PYY, the so-called gut peptides after LSG. It was hypothesized that the faster delivery of nutrients to the distal intestinal tract postoperatively may provoke increased the postprandial release of the gut peptides contributing to the improvement of glucose control as well as to the reduction of food intake and subsequently body weight [34].

The esophageal motility was assessed in several studies. The limitations of the studies include the small sample size. The data remain debatable. The variability in the outcomes of esophageal motility may be attributable to the timing of postoperative follow-up, the variability of surgical techniques, the different bougie sizes, and the different dissections from pylorus, thus creating a sleeve with variable distensibility and intraluminal pressure with, in part, the preservation of the antrum. The percentage of peristaltic normal contractions increased postoperatively and is not statistically significant at 3 months [11] but statistically significant later [5]. It seems that the motility of the body of the esophagus was normalized. Perhaps, this might be attributable to the reduced intrabdominal pressure due to weight loss. An increase in the esophageal acidification was reported in few studies [9, 11]. Del Genio et al. pointed out a decrease in the esophageal transit after LSG in impedance study. Furthermore, the authors explained the reflux episodes as a consequence of retrograde movements into the esophagus [9].

Gastric motility emerges as the role of the stomach as an endocrine organ. Gastric motility acts as a central mediator of hunger, satiation, and satiety. Gastric emptying plays a key role in regulating food intake. Gastric distention acts a satiety signal to inhibit food intake [35]. During food intake, it is the gastric distention and gastric accommodation that regulate satiation in a manner. After food intake, when the stomach empties gradually, it is the gastric emptying and the intestinal exposure of the nutrients that play a key role, while the role of gastric distention follows. It seems that gastric accommodation and gastric emptying are implicated in the regulation of gastric distention and intestinal exposure of nutrients, thus control satiation and satiety. The correlations between gastric accommodation, gastric emptying, and body weight suggest that gastric motility may also affect the long-term regulation of body weight [36].

A number of studies assessed gastric motility. Baumann et al. showed that the sleeve was completely motionless without any coordinate peristalsis, while the antrum had faster peristaltic folds, concluding that gastric emptying is directly linked to the function of the antrum when the antrum is preserved in LSG [23]. It seems that the resection of the normal gastric pacemaker during LSG had as a result the acute gastric slow-wave quiescence or the generation of distal ectopic pacemakers accompanied by a markedly increased propagation velocity. It seems that the sleeve affects the electrophysiology of the stomach. Further studies are needed to support this.

There was a consensus except for one study that gastric emptying was accelerated. The rapid gastric emptying was also strengthened by the observation of our team that a significant proportion of patients experienced dumping syndrome upon provocation at six weeks and 6 and 12 months after LSG. These symptoms included both early and late symptoms suggesting that LSG may lead to changes in eating patterns after LSG, especially in sweeters [37, 38]. Only one study showed no effect on gastric emptying. In this study, the follow-up was done in 3 months, and the created sleeve was performed using a bougie 48F at a distance of 6 cm from the pylorus [31]. Therefore, the sleeve might be large enough without increasing its intraluminal pressure.

There is still a debate concerning the maintenance of the antrum in order to avoid interference with the gastric physiology or its resection to increase the restrictive mechanism. Abdallah et al., who compared groups with resections at 2 cm versus 6 cm distances from the pylorus, showed that there was statistically significant excess weight loss between the groups, concluding that increasing the size of the resected antrum was associated with better weight loss, without increasing significantly the rate of complications [39]. Similarly, Obeidat et al. showed that patients with resection at 2 cm distance from the pylorus experienced statistically significant better maintained weight loss than did patients with resection at 6 cm [40]. On the contrary, ElGeidie et al. found no statistical differences regarding weight loss at the groups at 2 cm and 6 cm from the pylorus [41]. Vives et al. showed that gastric emptying was faster in the group with the resection at 3 cm from the pylorus compared to that at 8 cm from the pylorus at 6 and 12 months postoperatively [14]. Unlike the previous, Fallahat et al. compared the gastric emptying in patients at 4 cm and 7 cm distance from pylorus 3 months postoperatively. The authors showed that resection at 4 cm from the pylorus were associated with delayed gastric emptying, and resection at 7 cm from the pylorus, with accelerated gastric emptying. The authors speculated that resection at 4 cm from the pylorus had as a consequence neural innervations of the antrum being abolished, contributing to slower emptying. These patients experienced nausea, vomiting, and poor appetite. On the other hand, the resection at 7 cm from the pylorus preserved the contractility of the antrum, leading to rapid gastric emptying due to the absence of redistribution process since the body and fundus were excised. These patients complained of dumping-like symptoms and had more frequent meals [42]. Further prospective randomized trials are needed to compare the motility changes and clinical symptoms in patients with the preservation or not of antrum.

There is scant information concerning the possible explanation of how these motility alterations may affect clinical symptoms or explain the underlying mechanisms of action. It seems that the technique is not standardized, and different sleeves are created. Thus, different residual gastric volumes are produced. Since now, no clear association was found between gastric volume and weight loss. Researchers suggest that the physiological changes and not the size of the sleeve are responsible as mechanisms of action. What is known is that LSG changes the profile of gut hormones. Sista et al. showed that the rapid gastric emptying was correlated with the increased production of GLP-1 in the distal bowel [16]. Burgerhart et al. associated postprandial symptoms with gastric emptying. No difference on gastric-emptying characteristics was found between patients with low or high postprandial symptoms [24]. Pomerri et al. found that patients presenting with a rapid gastroduodenal transit experienced better weight loss than patients presenting with a slow voiding rate [18]. It seems that little is known regarding the underlining mechanisms by which LSG controls appetite and food intake. Since the data are restricted to medium term, it would be interesting to see if the sleeve behaves differently in the long term and how weight regain is explained in some patients.

Bowel motility was the least studied. It seems that the food reaches the terminal ileum faster but arrives at the cecum later. Thus, the contact of the food with the area of terminal ileum is extended. Perhaps, this altered interaction of food with the gastrointestinal tract may be a key component to explain the neurohormonal changes via the stimulation of intestinal L cells producing incretions and understanding the underlining mechanisms which improve the metabolic profile of the patients.

The limitations of the review include the lack of standardization of the surgical technique, the different follow-up timings, the different measured outcomes, the small sample siz,e and the lack of available long-term data beyond four years.

Further pathophysiological studies are needed to investigate the exact correlation of the motility parameters with the clinical symptoms and gut peptide alterations and potential hormonal interactions between gastrointestinal tract and brain.

## 5. Conclusion

Laparoscopic sleeve gastrectomy has impacts on the gastrointestinal motility. The data remain debatable for esophageal motility. The stomach and small bowel motilities were accelerated, while the initiation of cecal filling and the ileocecal valve transit was delayed. Further pathophysiological studies are needed to investigate the exact correlation of the motility parameters with the clinical symptoms and gut peptide alterations.

## Figures and Tables

**Table 1:** Studies assessing esophageal motility.

No.	Author, year of publication, country	Type of study	Patients	Method of assessment/follow-up	Distance from pylorus/bougie size	Main findings of the study
1	Sioka, E. et al., 2017 [5], Greece	Prospective study	18	Esophageal manometry preoperatively and at median interval of 7 months	5 cm/36F	The lower esophageal sphincter (LES) total length decreased postoperatively (*p* = 0.002). The resting and residual pressures tended to decrease postoperatively (mean difference [95% confidence interval]: −4 [−8.3/0.2] mmHg, *p* = 0.060; −1.4 [−3/0.1] mmHg, *p* = 0.071, resp.). The amplitude pressure decreased from 95.7 ± 37.3 to 69.8 ± 26.3 mmHg at the upper border of LES (*p* = 0.014) and tended to decrease at the distal esophagus from 128.5 ± 30.1 to 112.1 ± 35.4 mmHg (*p* = 0.06) and midesophagus from 72.7 ± 34.5 to 49.4 ± 16.7 mmHg (*p* = 0.006). The peristaltic normal swallow percentage increased from 47.2 ± 36.8 to 82.8 ± 28% (*p* = 0.003). The postoperative regurgitation was strongly negatively correlated with LES total length (Spearman's *r* = −0.670). When groups were compared according to heartburn status, the statistical significance was observed between the groups of improvement and deterioration regarding postoperative residual pressure and postoperative relaxation (*p* < 0.002 and *p* < 0.002, resp.). With regard to regurgitation status, there was statistically significant difference between groups regarding preoperative amplitude pressure at the upper border of LES (*p* < 0.056). Patients developed decreased LES length and weakened LES pressure after LSG. Esophageal body peristalsis was also affected in terms of decreased amplitude pressure, especially at the upper border of LES. Nevertheless, body peristalsis was normalized postoperatively. LSG might not deteriorate heartburn. Regurgitation might increase following LSG due to shortening of LES length, particularly in patients with range of preoperative amplitude pressure at the upper border of LES of 38.9–92.6 mmHg.

2	Mion, F. et al., 2016 [7], France	Retrospective study	53	High-resolution impedance manometry Sleeve volume and diameter with CT scan Median follow-up at 11 months (1–50)	NR/NR	The increased intragastric pressure occurred very frequently in patients after SG (77%) and was not associated with any upper GI symptoms, specific esophageal manometric profile, or impedance reflux. Impedance reflux episodes were also frequently observed after SG (52%): they were significantly associated with gastroesophageal reflux (GER) symptoms and ineffective esophageal motility. The sleeve volume and diameters were also significantly smaller in patients with impedance reflux episodes (*p* < 0.01). SG significantly modified esophagogastric motility. The IIGP is frequent, not correlated to symptoms and should be regarded as a HRIM marker of SG. The impedance reflux episodes were also frequent, associated with GER symptoms and esophageal dysmotility. HRIM may thus have a clinical impact on the management of patients with upper GI symptoms after SG.

3	Rebecchi, F. et al., 2014 [8], Italy	Prospective clinical study	65	Clinically validated questionnaire, upper endoscopy, esophageal manometry, and 24-hour pH monitoring before and 24 months after LSG.	6 cm/36F	On the basis of preoperative 24-hour pH monitoring, patients were divided into group A (pathologic, *n* = 28) and group B (normal, *n* = 37). The symptoms improved in group A, with the gastroesophageal reflux disease symptom assessment scale score decreasing from 53.1 ± 10.5 to 13.1 ± 3.5 (*p* < 0.001). The DeMeester score and total acid exposure (% pH < 4) decreased in group A patients (DeMeester score from 39.5 ± 16.5 to 10.6 ± 5.8, *p* < 0.001; % pH < 4 from 10.2 ± 3.7 to 4.2 ± 2.6, *p* < 0.001). Real de novo GERD occurred in 5.4% of group B patients. No significant changes in the lower esophageal sphincter pressure and esophageal peristalsis amplitude were found in both groups. LSG improves symptoms and controls reflux in most morbidly obese patients with preoperative GERD. In obese patients without preoperative evidence of GERD, the occurrence of de novo reflux is uncommon. Therefore, LSG should be considered as an effective option for the surgical treatment of obese patients with GERD.

4	Gorodner, V. et al., 2015 [10], Argentina	Prospective study	14	Esophageal manometry (EM) and 24 h pH monitoring before and 1 year after LSG.	6 cm/36F	The lower esophageal sphincter (LES) length increased from 2.7 to 3.2 cm (*p* = NS), and LES pressure decreased from 17.1 to 12.4 mmHg (*p* ≤ 0.05). Preoperatively, LES was normotensive in 13 (93%) patients; postoperatively, LES was normal in 10 (71%) (*p* = NS). After LSG, the LESP significantly decreased

5	Burgerhart, J. S. et al., 2014 [11], Netherlands	Prospective study	20	Esophageal function tests (high-resolution manometry (HRM), 24-h pH/impedance metry) before and 3 months after LSG	6 cm/34F	Esophageal acid exposure significantly increased after sleeve gastrectomy: upright from 5.1 ± 4.4 to 12.6 ± 9.8% (*p* = 0.003), supine from 1.4 ± 2.4 to 11 ± 15% (*p* = 0.003) and total acid exposure from 4.1 ± 3.5 to 12 ± 10.4% (*p* = 0.004). The percentage of normal peristaltic contractions remained unchanged, but the distal contractile integral decreased after LSG from 2006.0 ± 1806.3 to 1537.4 ± 1671.8 mmHg · cm · s (*p* = 0.01). The lower esophageal sphincter (LES) pressure decreased from 18.3 ± 9.2 to 11.0 ± 7.0 mmHg (*p* = 0.02). After LSG, the patients have significantly higher esophageal acid exposure, which may well be due to a decrease in the LES resting pressure following the procedure.

6	Del Genio, J. et al., 2014 [9], Italy	Prospective study	25	High-resolution impedance manometry (HRiM) and combined 24 h pH and multichannel intraluminal impedance (MII-pH). Median follow-up at 13 months	NR/40F	Unchanged LES function, increased ineffective peristalsis, and incomplete bolus transit. MII-pH showed an increase of both acid exposure of the esophagus and number of nonacid reflux events in postprandial periods. Laparoscopic SG is an effective restrictive procedure that creates delayed esophageal emptying without impairing LES function. A correctly fashioned sleeve does not induce de novo GERD. Retrograde movements and increased acid exposure are probably due to stasis and postprandial regurgitation.

7	Kleidi, E. et al., 2013 [6], Greece	Prospective study	23	Esophageal manometry preoperatively and 6 weeks postoperatively	3-4 cm/34F	The LES total and abdominal lengths increased significantly postoperatively, whereas the contraction amplitude in the lower esophagus decreased. There was an increase in reflux symptoms postoperatively (*p* < 0.009). The approximation of the angle of His mostly from the operating surgeon resulted in an increased abdominal LES length (*p* < 0.01). The presence of esophageal tissue in the specimen correlated with the increased total GERD score (*p* < 0.05). LSG weakens the contraction amplitude of the lower esophagus, which may contribute to postoperative reflux deterioration. It also increases the total and the abdominal lengths of the LES, especially when the angle of His is mostly approximated. However, if this approximation leads to esophageal tissue excision, the reflux is again aggravated. Thus, stapling too close to the angle of His should be done cautiously.

8	Petersen, W. V. et al., 2012 [12], Germany	Prospective study	37 Group I (BMI = 19–25) 20 (control group) Group II (BMI = 42–65) 20 patients (8 months) Group III (BMI = 37–57) 17 patients (6 days)	Esophageal manometry	2 cm/35 Ch	Postoperatively, the LESP increased significantly, namely, from preoperative 8.4 to 21.2 mmHg in group II and from 11 to 24 mmHg (*p* < 0.0001) in group III. The tubular esophageal motility profits from LSG. The LSG significantly increased the lower esophageal pressure independent of weight loss after LSG and may protect obese patients from gastroesophageal reflux.

9	Braghetto, I. et al., 2010 [13], Chile	Prospective study	20	Esophageal manometry preoperatively, 6 months postoperatively	2 cm/32F	Preoperative mean LESP was 14.2 ± 5.8 mmHg. The postoperative manometry decreased in 17/20 (85%), with a mean value of 11.2 ± 5.7 mmHg (*p* = 0.01). Seven of them presented LESP of <12 mmHg and ten patients presented LESP of <6 mmHg after the operation. Furthermore, the abdominal length and total length of the high pressure zone at the esophagogastric junction were affected. A sleeve gastrectomy produces an important decrease in LES pressure, which can, in turn, cause the appearance of reflux symptoms and esophagitis after the operation due to a partial resection of the sling fibers during the gastrectomy.

**Table 2:** Studies assessing stomach motility.

	Author, year of publication, country	Type of study	Patients (*N*)	Method of assessment/follow-up points	Bougie size/distance from pylorus	Main findings of the study
1	Vives, M. et al., 2017 [14], Spain	Prospective randomized study	60 (30 patients with the section at 3 cm and 30 patients with that at 8 cm from the pylorus	Gastric emptying by scintigraphy (*T*1/2 min), gastric volume by CT scan (cc) at 6 and 12 months	38F/3 cm/8 cm	Gastric emptying increases the speed significantly in both groups but is greater in the 3 cm group (*p* < 0.05). When dividing groups into type 2 diabetic patients and nondiabetic patients, the speed in nondiabetic patients is significantly higher for the 3 cm group. The residual volume increases significantly in both groups, and there are no differences between them. Gastric emptying is faster in patients with antrum resection. The distance does not influence the gastric emptying of diabetic patients.

2	Berry, R. et al., 2017 [28], New Zealand	Prospective study	8 (1 patient with chronic refux, nausea, and dysmotility)	Laparoscopic high-resolution (HR) electrical mapping before and after LSG	NR	The baseline activity showed exclusively normal propagation. Acutely after LSG, all patients developed either a distal unifocal ectopic pacemaker with retrograde propagation (50%) or bioelectrical quiescence (50%). The propagation velocity was abnormally rapid after LSG (12.5 ± 0.8 versus baseline 3.8 ± 0.8 mm s^−1^; *p* = 0.01), whereas the frequency and amplitude were unchanged (2.7 ± 0.3 versus 2.8 ± 0.3 cpm, *p* = 0.7; 1.7 ± 0.2 versus 1.6 ± 0.6 mV, *p* = 0.7). In the patient with chronic dysmotility after LSG, mapping also revealed a stable antral ectopic pacemaker with retrograde rapid propagation (12.6 ± 4.8 mm s^−1^). The resection of the gastric pacemaker during LSG acutely resulted in aberrant distal ectopic pacemaking or bioelectrical quiescence. Ectopic pacemaking can persist long after LSG, inducing chronic dysmotility. The clinical and therapeutic significance of these findings now require further investigation.

3	Sista, F. et al., 2017 [16], Italy	Prospective study	52	Gastric emptying scintigraphy for liquid and solid foods, before and 3 months after LSG.	36F/5 cm	After surgery, *T*_1/2_ was significantly accelerated: 15.2 ± 13 min and 33.5 ± 18 min in the L group and S group, respectively (*p* < .05). In both groups, GLP-1 plasma concentrations were increased at each blood sampling time: 2.91 ± 2.9 pg/mL, 3.06 ± 3.1 pg/mL, and 3.21 ± 2.6 pg/mL at 15, 30, and 60 minutes, respectively, (*p* < .05) for the L group and 2.72 ± 1.5 pg/mL, 2.89 ± 2.1 pg/mL, 2.93 ± 1.8 pg/mL, and 2.95 ± 1.9 pg/mL at 30, 60, 90, and 120 minutes, respectively, (*p* < .05) for the S group. After LSG, GLP-1 and %GR presented a negative linear correlation (*r*) at each blood sampling time in both groups. Rapid gastric emptying 3 months after LSG

4	Vigneshwaran, B. et al., 2016 [26], India	Prospective study	20 with T2DM and with a BMI of 30.0–35.0 kg/m^2^	The gastric emptying times were measured at baseline, 3 months, 6 months, 12 months, and 24 months after surgery.	36F/4 cm	There was a significant decrease in gastric emptying time. Accelerated gastric emptying

5	Mans, E. et al., 2015 [15], Spain	Prospective comparative study	Three groups were studied: morbidly obese patients (*n* = 16), morbidly obese patients who had had sleeve gastrectomy (*n* = 8), and nonobese patients (*n* = 16)	Gastric and gallbladder emptying	42F/5 cm	The antrum area during fasting in morbidly obese patients was statistically significantly larger than that in the nonobese and sleeve gastrectomy groups. Gastric emptying was accelerated in the sleeve gastrectomy group compared with the other 2 groups (which had very similar results). Gallbladder emptying was similar in the 3 groups. Gastric emptying was accelerated in the sleeve gastrectomy group compared with the other 2 groups (which had very similar results)

6	Kandeel, A. A. et al., 2015 [29], Egypt	Prospective study	40	Tc-sulfur colloid GE scintigraphy was performed on all patients submitted to LSG before and after surgery (1–4 weeks for liquids and 4–6 weeks for solids)	36F/3–4 cm	*T*1/2 was significantly enhanced after LSG compared with the baseline (25.3 ± 4.4 versus 11.8 ± 3.0 min for liquids and 74.9 ± 7.1 versus 28.4 ± 8.3 min for solids, resp., *p* < 0.001). The percentage of gastric retention in operated patients was significantly less than that at baseline for liquids at 15, 30, and 60 min (33.9 ± 5.6, 17.7 ± 3.9, and 7.5 ± 2.8% versus 69.4 ± 10.5, 55.6 ± 14.95, and 26.1 ± 4.7%, resp., *p* < 0.001), as well as for solids at 30, 60, 90, and 120 min (42.0 ± 11.1, 20.8 ± 6.1, 11.0 ± 5.9, and 3.8 ± 2.7% versus 79.9 ± 8.7, 67.4 ± 12.2, 37.0 ± 10.9%, and 13.8 ± 4.4%, resp., *p* < 0.001). The significant acceleration of GE of liquids and solids after LSG may have contributed to weight loss in the immediate postoperative period (4–6 weeks). It remains to be determined whether the weight loss will continue beyond that period.

7	Burgerhart, J. S. et al., 2015 [24], Netherlands	Prospective study	20	Gastric emptying study with solid and liquid meal components in the second year after LSG	34F/6 cm	The lag phase (solid) was 6.4 ± 4.5 min in group I and 7.3 ± 6.3 in group II (*p* = 0.94); *T*1/2 (solid) was 40.6 ± 10.0 min in group I and 34.4 ± 9.3 in group II (*p* = 0.27); the caloric emptying rate was 3.9 ± 0.6 kcal/min in group I and 3.9 ± 1.0 kcal/min in group II (*p* = 0.32). Patients with postprandial symptoms after LSG reported more symptoms during the gastric emptying study than did patients without symptoms. However, there was no difference between gastric emptying characteristics between both groups, suggesting that abnormal gastric emptying is not a major determinant of postprandial symptoms after LSG.

8	Melissas, J. et al., 2013 [21], Greece	Prospective study	21	The gastric transit times were studied with a gamma camera before and 4 months postoperatively	34F/5 cm	SG accelerates the gastric emptying of semisolids

9	Pilone, V. et al., 2013 [17], Italy	Prospective controlled randomized study	45 Group A exam before (A1) and after the operation (A2). Control group (Group B)	Gastric emptying scintigraphy 1 month preoperatively and 3 months postoperatively	34Ch/4–5 cm	The scintigraphic study showed a reduced half-life tracer (A1 versus A2: 80.4 ± 16.5 min versus 64.3 ± 22 min *p* = 0.06), without a significant difference. Comparing the two groups, no differences occurred before operation (B versus A1). The gastric emptying time resulted in a significant reduction in group A2 rather than in groups A1 and B. LSG reduces gastric emptying time

10	Michalsky, D. et al., 2013 [22], Czech Republic	Prospective randomized study	12	Group A antrum resection Group B Antrum preservation Gastric emptying scintigraphy before and 3 months postoperatively	42F/7cm	In the antrum resection group, the average time T1/2 declined from 57.5 to 32.25 min (*p* = 0.016) and average retention %GE dropped from 20.5 to 9.5% (*p* = 0.073). In the antrum resection group, an increase in gastric emptying postoperatively was noted. Complications such as failure of stomach evacuation were not observed in the RA group; even more radical resection of the pyloric antrum performed by LSG is possible without concerns of postoperative disorder of the stomach evacuation function

11	Parikh, M. et al., 2012 [25], USA	Data from an institutional review board-approved electronic registry	62	Gastroduodenal transit time (antrum to duodenum) was calculated from a postoperative day 1 esophagram. Postoperative esophagrams	40F/5–7 cm	The mean gastroduodenal transit time was 12.3 ± 19.8 s. Almost all patients (99%) had a transit time of less than 60 s. No correlation was found between gastroduodenal transit time and %EWL at 3, 6, or 12 months.

12	Baumann, T. et al., 2011 [23], Germany	Prospective pilot study	5	MRI 1 day before LSG and 6 days and 6 months after LSG	32F/5–6 cm	The dynamic analysis showed that antral propulsive peristalsis was preserved immediately after surgery and during follow-up, but fold speed increased significantly from 2.7 mm/s before LSG to 4.4 mm/s after 6 months. The sleeve itself remained without recognizable peristalsis in three patients and showed only uncoordinated or passive motion in two patients. Consequently, the fluid transport through the sleeve was markedly delayed, whereas the antrum showed accelerated propulsion with the emptying half-time decreasing from 16.5 min preoperatively to 7.9 min 6 months after surgery. The stomach is functionally divided into a sleeve without propulsive peristalsis and an accelerated antrum. Accelerated emptying seems to be caused by faster peristaltic folds.

13	Pomerri, F. et al., 2011 [18], Italy	Retrospective study	57	The size of the gastric fundus remaining after LSG and gastric voiding rate (fast/slow) by radiological upper gastrointestinal series (UGS) with a water-soluble contrast medium (CM).	NR/4–6 cm	Sleeve voiding was fast in 49 of 57 patients (85.96%) and slow in eight (14.03%). Patients showing a rapid gastroduodenal transit of the CM achieved a better weight loss than patients with a slow voiding rate.

14	Shah, S. et al., 2010 [27], India	Prospective controlled study	24 were lean controls (body mass index 22.2 ± 2.84 kg/m (2)), 20 were severely and morbidly obese patients with T2DM who had not undergone SG (body mass index 37.73 ± 5.35 kg/m (2)), and 23 were severely and morbidly obese patients with T2DM after SG.	Scintigraphic imaging with g-camera	NR	The gastric emptying half-time values were also significantly shorter (*p* < .05) in the post-SG (52.8 ± 13.5 minutes) than in the non-SG (73.7 ± 29.0 minutes) and control (72.8 ± 29.6 minutes) groups decreased gastric emptying half-time after SG

15	Braghetto, I. et al., 2009 [30], Chile	Prospective study	20 obese submitted to LSG 18 normal subjects	Gastric emptying of liquids and solids was measured by scintigraphic technique 3 months postoperatively.	32F/2 cm	In the group of operated patients, 70% of them (*n* = 14) presented accelerated emptying for liquids and 75% (*n* = 15) for solids compared to 22.2% and 27.7%, respectively, in the control group. The half-time of gastric emptying (T(1/2)) in patients submitted to SG both for liquids and solids were significantly more accelerated compared to the control group (34.9 ± 24.6 versus 13.6 ± 11.9 min for liquids and 78 ± 15.01 versus 38.3 ± 18.77 min for solids; *p* < 0.01). The gastric emptying for liquids expressed as the percentages of retention at 20, 30, and 60 min were 30.0 ± 0.25%, 15.4 ± 0.18%, and 5.7 ± 0.10%, respectively, in operated patients, significantly less than the control subjects (*p* < 0.001). For solids, the percentage of retention at 60, 90, and 120 min was 56 +/− 28%, 34 +/− 22%, and 12 +/− 8%, respectively, for controls, while it was 25.3 +/− 0.20%, 9 +/− 0.12%, and 3 +/− 0.05%, respectively, in operated patients (*p* < 001). Gastric emptying after SG is accelerated either for liquids as well as for solids in the majority of patients.

16	Bernstine H et al., 2009 [31], Israel	Prospective study	21	Gastric emptying scintigraphy of semisolids was performed before and 3 months after LSG	48F/6 cm	The mean T 1/2 raw data were 62.39 ± 19.83 and 56.79 ± 18.72 min (*p* = 0.36, *t* = −0.92, NS) before and 3 months after LSG, respectively. The T 1/2 linear was 103.64 ± 9.82 and 106.92 ± 14.55, (*p* = 0.43, *t* = −0.43, NS), and the linear fit slope 0.48 ± 0.04 and 0.47 ± 0.05 (*p* = 0.48, *t* = 0.7, NS). LSG with antrum preservation as performed in this series has no effect on gastric emptying.

17	Melissas, J. et al., 2008 [20], Greece	Prospective study	14	Nine patients underwent gastric emptying studies, using radioisotopic technique before, 6 months, and 24 months after the operation. The remaining five patients underwent gastric emptying studies, 6 months and 24 months after the operation. Scintigraphic imaging was performed with a *γ*-camera	NR	In the nine patients who underwent gastric emptying studies preoperatively and 6 and 24 months postoperatively, the T-lag phase duration significantly decreased, following the SG, from 17.30 (range 15.50–20.90) min, to 12.50 (range 9.20–18.00) min at 6 months and 12.16 (range 10.90–20.00) min at 24 months postoperatively (*p* < 0.05) The gastric emptying half time (*T*1/2) accelerated significantly postoperatively from 86.50 (range 77.50–104.60) min, to 62.50 (range 46.30–80.00) min at 6 months and 60.80 (range 54.80–100.00) min at 24 months after SG (*p* < 0.05). The percentage of gastric emptying (%GE) increased significantly postoperatively, from 52 (range 43–58) % to 72 (range 57–97) % at 6 months and 74 (range 45–82) % at 24 months, following SG (*P* < 0.05). No differences in gastric emptying were observed, when values at 24 months were compared to those at 6 months postoperatively. When the whole group of 14 patients was studied, there were also no significant changes in T-lag, T1/2 and %GE between 6 and 24 months postoperatively. Constant effect of SG in the acceleration of gastric emptying of solids, which occurs faster, not only in short but also in long-term postoperatively

18	Melissas, J. et al., 2007 [19], Greece	Prospective study	23	The scintigraphic measurement of the gastric emptying of a solid meal preoperatively and 6 months postoperatively. Gastric emptying studies using radioisotopic technique before and 6 months after the operation.	34F/7 cm	Although the meal size was drastically reduced, the meal frequency increased postoperatively in 12 patients (52.2%). Only 5 patients (21.8%) reported occasional vomiting after meals following SG. The gastric emptying half-time (T1/2) accelerated (47.6 ± 23.2 versus 94.3 ± 15.4, *p* < 0.01), and the T-lag phase duration decreased (9.5 ± 2 min versus 19.2 ± 2 min, *p* < 0.05) postoperatively. The percentage of the meal emptied from the stomach 90 min after consumption increased significantly after SG (75.4 ± 14.9% versus 49.2 ± 8.7%, *p* < 0.01); the stomach empties its contents rapidly into the small intestine and symptoms of vomiting after eating (characteristic of restrictive procedures) are either absent or very mild

NR: not reported.

**Table 3:** Studies assessing bowel motility.

	Author, year of publication, country	Type of study	Patients (*N*)	Method of assessment	Study main finding
1	Trung, V. N. et al., 2013 [32], Japan	Prospective study	12	Intestinal motility during OGTT was assessed using cine MRI before and 3 months postoperatively	LSG leads to accelerated intestinal motility and reduced intestinal transit time

2	Melissas, J. et al., 2013 [21], Greece	Prospective study	21	g-Camera before and 4 months postoperatively	LSG accelerates the small bowel transit of semisolids. In addition, it delays the initiation of cecal filling and T ICVt.

3	Shah, S. et al., 2010 [27], India	Prospective controlled study	67 Controls: 24 Morbidly obese patients with T2DM not undergone SG: 20 Morbidly obese patients with T2DM after SG: 23	Scintigraphic imaging	Decreased small bowel transit time after SG
